# miRBind: A Deep Learning Method for miRNA Binding Classification

**DOI:** 10.3390/genes13122323

**Published:** 2022-12-09

**Authors:** Eva Klimentová, Václav Hejret, Ján Krčmář, Katarína Grešová, Ilektra-Chara Giassa, Panagiotis Alexiou

**Affiliations:** 1 Central European Institute of Technology (CEITEC), Masaryk University, 60177 Brno, Czech Republic; 2 Faculty of Science, National Centre for Biomolecular Research, Masaryk University, 61137 Brno, Czech Republic; 3 Faculty of Informatics, Masaryk University, 60200 Brno, Czech Republic

**Keywords:** miRNA:target prediction, miRNA binding, CLASH, convolutional neural network

## Abstract

The binding of microRNAs (miRNAs) to their target sites is a complex process, mediated by the Argonaute (Ago) family of proteins. The prediction of miRNA:target site binding is an important first step for any miRNA target prediction algorithm. To date, the potential for miRNA:target site binding is evaluated using either co-folding free energy measures or heuristic approaches, based on the identification of binding ‘seeds’, i.e., continuous stretches of binding corresponding to specific parts of the miRNA. The limitations of both these families of methods have produced generations of miRNA target prediction algorithms that are primarily focused on ‘canonical’ seed targets, even though unbiased experimental methods have shown that only approximately half of in vivo miRNA targets are ‘canonical’. Herein, we present miRBind, a deep learning method and web server that can be used to accurately predict the potential of miRNA:target site binding. We trained our method using seed-agnostic experimental data and show that our method outperforms both seed-based approaches and co-fold free energy approaches. The full code for the development of miRBind and a freely accessible web server are freely available.

## 1. Introduction

miRNAs are endogenous small (~17–25 nucleotides long) ncRNAs that negatively regulate gene expression at the level of messenger RNA (mRNA) [1]. The first miRNA (lin-4) was discovered in *Caenorhabditis elegans* in 1993 [2,3]; in humans, the first miRNA that was discovered is let-7, first identified in 2000 in *C. elegans* [4]. To date, 2654 mature human miRNAs have been deposited in the miRbase [5] miRNA database. miRNAs are processed from hairpin-containing primary transcripts (pri-miRNAs); they are subsequently processed into precursor miRNAs (pre-miRNAs) [6], exported to the cytoplasm [7], and cleaved into small double-stranded RNAs [8,9]. The mature miRNA duplex is then loaded into an argonaute (AGO) protein to form a miRNA-induced silencing complex (miRISC). Mature miRNAs interact with the AGO proteins and guide them, via base pairing, toward target RNAs. Such targeting may lead to translational repression and deadenylation-induced mRNA degradation [10,11]. Each miRNA can have thousands of binding sites on the transcriptome, and an mRNA can contain dozens of potential miRNA binding sites [12]. Animal miRNAs occasionally show extensive, but more often only partial, complementarity with their target sites [13,14]. The 5′ end of the miRNA, and especially the hexamer-spanning nucleotides 2–8, were identified very early on as being important for miRNA target recognition and were termed the ‘seed’ region [15]. Target recognition is primarily achieved via base pairing that involves the seed region [16]; however, seed pairing is not always sufficient for functional target interactions, and additional interactions with the miRNA 3′ end may be necessary for specific targeting [17]. Targeting can also be facilitated by additional sequence elements, such as an unpaired adenosine in the 5′-end of the miRNA [18]. An estimated quantification of non-canonical miRNA binding sites calculates that approximately 60% of all identified interactions are based on non-canonical seeds [19,20].

Early approaches to miRNA target prediction implemented additional features, such as the evolutionary conservation levels of targets, positioning of target sites on 3′-UTRs, nucleotide content of targets, and others [16]. Another family of miRNA target prediction methods utilized alignment or co-fold methodologies, ignoring the ‘seed’ region [21,22,23]. In these approaches, an idealized structure is calculated, based on the affinity of the miRNA sequence to the putative target sequence; then, measures such as alignment score or the free energy of binding of the two molecules are used to score binding potential. When the first high-throughput miRNA targeting datasets became available [24,25], ‘seed’-based approaches appeared to outperform the ‘folding’-based methods on all benchmarks of precision and sensitivity [26]. The following years saw a wealth of high-throughput miRNA targeting data being produced, utilizing techniques in the CLIP-Seq (cross-linking immunoprecipitation sequencing) [27] family, which identified thousands of miRNA-target mRNA pairs [28]. CLIP is based on the stabilization of protein-RNA complexes in their cellular environment by UV cross-linking, the immunoprecipitation of ribonucleoprotein complexes (RNPs), and the isolation and sequencing of bound RNAs. An important limitation of such techniques is that they do not produce specific miRNA:target site pairs. Instead, they produce peaks of Ago protein-binding to which miRNAs need to be assigned, often using a miRNA target prediction program that utilizes the ‘seed’ heuristic. Even though more functional non-canonical ‘seed’-binding sites are being continuously discovered, they remain underrepresented by all miRNA prediction programs and databases of validated miRNA targets.

CLASH (cross-linking, ligation, and sequencing of hybrids) is the first reported high-throughput method for the direct identification of RNA–RNA interactions [18] The CLASH method allows for the precise mapping of interactions for which the downstream consequences are unknown and/or difficult to measure. Because cross-linking is performed in living cells, the dynamic state of the RNA interactome can be probed as a function of physiological conditions. It offers two types of information: precise AGO-binding sites on RNAs (similar to CLIP methods), and RNA–RNA hybrids that are formed within the AGO RNA-binding pocket [29]. In 2013, the first unbiased experimental method for the identification of Ago1 miRNA binding sites in cell culture was performed, using the CLASH technique. The study showed that approximately 60% of identified miRNA:target duplexes contain a non-canonical ‘seed’, while 18% of targets show binding in the 3′ end of the miRNA without any ‘seed’ binding [20]. In this seminal paper, the authors used a miRNA:target site ‘co-fold free energy’ approach to predict the type of binding. Subsequent studies utilized CLASH and CLEAR (covalent ligation of endogenous Argonaute-bound RNAs) [30] techniques to identify more miRNA:target site pairs, and solidify the abundance and functionality of ‘non-seed’ target sites. Despite this well-documented functionality of the ‘non-seed’ target sites, the vast majority of miRNA target prediction programs today still use the ‘seed’ heuristic as a first filtering step [31].

As the studies on miRNA:target binding rules present researchers with new challenges, advances in computational approaches, including machine learning (ML), have gained great significance. ML techniques have been applied to predict miRNA targets and there have been several reviews covering advancements in the field [32,33,34]; however, ML performance largely depends on the user-defined variables that were selected to train the model. Among the early adopters of ML approaches for miRNA target prediction are miRanda-mirSVR [35], DIANA-micro-T-CDS [36,37,38], mirTarget2 [39], TargetMiner [40], and SVMicrO [41]. miRanda-mirSVR incorporates support vector regression (SVR), while DIANA-micro-T-CDS utilizes generalized linear models (GLM); the rest of the mentioned classifiers are based on support vector machines (SVM). Deep learning (DL), an emerging field of ML, solves the issue of handcrafted features by embedding the computation of these features into the ML model itself [42]. Thus, DL is highly appropriate for uncovering the miRNA binding rules, where clear rules or features are unknown. In the past decade, Deep Neural Networks (DNN) [43] have found extensive use in many scientific fields, including bioinformatics [44]. Convolutional neural networks (CNNs) are a subtype of DNN that utilize several layers of convolutional neurons to learn increasingly complicated representations of input data. Input data is provided in a raw format, allowing the CNN to learn what patterns in the input data are important for a specific task. Selecting an appropriate training dataset and suitable evaluation metrics are of pivotal importance when building an effective DL model.

ResNet [45] is another type of DNN that uses an innovative architecture that enables the training of very deep models. They address the problem of vanishing gradient and help with optimization by utilizing a special kind of residual block that adds a skip connection. ResNet is used as a backbone for many computer vision tasks.

An important factor to be addressed by the miRNA target prediction methods that are based on classifiers is class imbalance. Each mRNA can be regulated by dozens of miRNAs and each miRNA has thousands of potential binding sites on the transcriptome [12]. The imbalance between the number of actual, experimentally verified binding sites (positive class) and all other regions on the transcriptome (negative class) has a significant, deteriorating effect on the performance of the prediction methods.

Since class imbalance is identified as the leading challenge influencing the performance of any prediction model, we propose miRBind, a novel method dealing with this issue, which is based on targeted sample selection and subsequent label smoothing. miRBind is a ResNet-based method trained on unbiased miRNA:target site CLASH data [20] and is shown to consistently outperform both ‘seed’ and ‘co-fold’ approaches in a binding-site classification task. We also provide an alternative CNN approach, a six-convolutional-layer network, with comparable performance. For ease of access, we provide a standalone Python program, as well as a freely available web server with a user-friendly interface (GitHub repository https://github.com/ML-Bioinfo-CEITEC/miRBind accessed on 28 November 2022, web server https://ml-bioinfo-ceitec.github.io/miRBind/ accessed on 28 November 2022).

## 2. Materials and Methods

### 2.1. Data Preparation

We retrieved the positive miRNA:target interaction dataset from the original CLASH study published by Helwak et al. [20]. We produced the negative dataset by randomly matching the miRNAs and target sites found in the positive dataset. A detailed explanation of the dataset’s production follows.

The positive dataset was constructed from the miRNA:target interactions identified by Helwak et al. in 2013, via their CLASH [20] experiment. The interacting miRNA:target pairs were downloaded and processed by standardizing the length of the miRNA sequences to 20 nt, anchored at the 5′ end of the miRNA, and centering and resizing the target coordinates to the window length of 50 bp. The resized target sequences were extracted using bedtools and the hyb reference [46] (https://github.com/gkudla/hyb/tree/master/data/db (accessed on 14 November 2021)). These processed miRNA:target pairs are known as the positive dataset. The positive dataset was divided into training, testing, and validation sets containing 15,392, 2000, and 1000 miRNA:target pairs.

Negative sets were constructed by excluding the interacting miRNA:mRNA partners provided by the 2013 CLASH experiment. More specifically, we created the negative datasets by matching real target sequences with random miRNAs from the same experiment, excluding the original positive set. We have elected to adopt this approach instead of choosing other parts of the genome/transcriptome as ‘fake’ targets, so as to avoid introducing any nucleotide content or other biases. We believe that shuffling the miRNA:target pairs is a fair and realistic approach that matches the way in which miRNAs may be assigned to Ago-CLIP peaks. To create the final datasets, positive and negative dataset parts were combined for the training and validation sets for the positive:negative ratios of 1:1, 1:10, 1:20, and 1:100. The CNN models were trained on all ratios, while miRBind was trained on the 1:1, 1:10 and 1:20 ratios, and DNABERT was fine-tuned on the 1:1 and 1:10 training sets. Testing sets were constructed for the positive:negative ratios of 1:1, 1:10 and 1:100.

**Figure 1:**
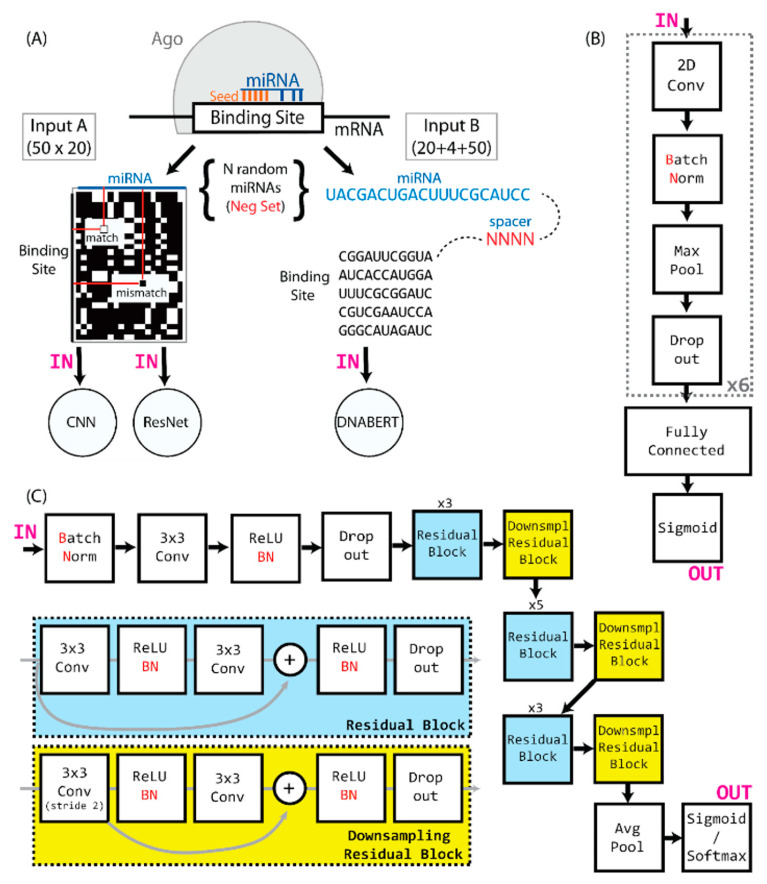
(**A**) Ago binding sites that are identified by CLASH are converted to two-dimensional arrays of matching and mismatching nucleotides, based on the miRNA and target sequence, and then used as input to the CNN and ResNet. The input to the DNABERT is constructed by interlaying the miRNA and target sequences with a 4-nt spacer. (**B**) A compact representation of the CNN network architecture. (**C**) A representation of the miRBind architecture.

To avoid any potential target sequence or experimental biases, we elected to completely hide the sequences from the convolutional neural network during training, instead using a two-dimensional representation of miRNA and putative target sequence, in which any Watson–Crick binding nucleotide pair is represented by 1, and any non-binding pair by 0. This creates a 20 × 50 two-dimensional matrix of 1 s and 0 s, which is the input for our training method (Figure 1A).

### 2.2. Independent Chimeric Read Dataset (miRNA eCLIP)

We have produced a novel evaluation dataset based on miRNA:target gene interactions, using a novel miR-eCLIP method [47]. The experimental process was performed on our behalf by Eclipse Bioinnovations (miR-eCLIP) and all primary data is deposited on NCBI:GEO with the accession (GSE218466). Briefly, Ago2-associated miRNA:target chimeric pairs were identified by: (a) removing the sequenced reads that fully map (over 85% of read-length) to the reference genome; (b) identifying reads that partially map on miRNA annotated in miRbase on a mature miRNA collection (Release 22.1), but not in databases of rRNAs, tRNAs, yRNAs, and vRNAs (annotations from NCBI [48], Ensembl [49], and the UCSC genome browser [50]—a full list of annotations can be found in the pipeline documentation (see below); (c) unmapped (soft-clipped) parts of these miRNA reads were remapped on the reference genome and annotated on known transcripts from Ensembl. Multiple overlapping genomic alignments mapping to the same miRNA were collapsed into single interactions and were extended to a 50 nt length around the center of the genomic alignment, to make sure that the whole binding site was captured. In all, we produced 477 such high-confidence miRNA:target gene interactions. Negative interactions were again produced by randomly shuffling the miRNA and target sequences found in the positive sample. The fully documented and publicly available pipeline for chimeric interaction detection is available at https://github.com/ML-Bioinfo-CEITEC/HybriDetector/ (accessed on 28 November 2022).

### 2.3. Benchmarking Approaches

There are certain factors that should be considered when selecting a method for benchmarking comparison: (i) the method must be able to predict binding-site affinity (i.e., to give a score representing the potential of a microRNA to bind to a target site), or, at the minimum, binary classification of the binding site (e.g., seed binding); (ii) the method must work directly on sequences and using sequences only (microRNA, target site); (iii) the method must have an implementation method that is relatively easy to use (standalone program). To this end, we excluded from our study the following prediction methods: (a) target prediction methods that aim to predict microRNA:target gene interactions, (b) methods based on a combination of multiple inputs (e.g., evolutionary conservation of the target), and (c) methods that do not have the full implementation to predict target sites. It is not feasible to re-implement those theoretical methods that lack functional code.

#### 2.3.1. CNN Approach

We utilized a convolutional neural network consisting of multiple layered blocks, composed of a convolutional layer, leaky ReLU, batch normalization, pooling, and a dropout layer. The output of the last dropout layer is flattened and connected to the layered blocks of dense, leaky ReLU, with batch normalization and a dropout layer. The last layer is formed of a single neuron with a sigmoid activation function that outputs the probability of input miRNA:target site binding. A schematic illustration of the network architecture can be found in Figure 1B. The network was compiled with the Adam optimizer and utilized the binary cross-entropy loss function. The models were trained over 10 epochs, with a batch size of 32. To find the best set of parameters to use, a hyperparameter search was performed separately for all positive:negative ratios (1:1, 1:10, 1:20, and 1:100), using the training set for model training and the evaluation set for comparison. The best model consisted of 6 blocks with convolutional layers, followed by 2 blocks with dense layers (Figure 1B). Convolutional layers had kernels sized 5 × 5, the dropout rate in the dropout layers was 0.3, and the learning rate was 0.00152. Through a subsequent evaluation, we concluded that the best-performing model is the one trained on the 1:10 training set.

#### 2.3.2. DNABERT

DNABERT [51] is a previously published transformer-based model that has achieved superior performance across various downstream DNA sequence prediction tasks. DNABERT uses tokenized k-mer sequences as its input, which also contains 3 additional tokens, and it can be fine-tuned for multiple purposes. Since the input to DNABERT is a set of sequences, we converted each miRNA:target pair into a single sequence, in which miRNA and target sequences were interlaid with 4 N nucleotides, as depicted in Figure 1A. We used a DNABERT model that was pretrained on the 6-mers and finetuned it on the 1:1 and 1:10 training sets, using a batch size of 64, 4 gradient accumulation steps, a learning rate of $2\times 10^{-4}$, a weight decay of 0.01, and early stopping with patience of 5.

#### 2.3.3. miRBind

The architecture of miRBind is a modified version of ResNet [45], in which the initial 7 × 7 convolution and pooling layers are removed and the number of residual blocks is optimized, as shown in Figure 1C. Since our input size is a 50 × 20 matrix, the initial layer and pooling, if left in place, would reduce the input for the subsequent layers to 12 × 5, thus not allowing the network to learn from the data. The miRBind architecture is illustrated in Figure 1C.

To address the imbalance between positive and negative classes in our 1:100 dataset, we developed a novel approach called instance hardness-based label smoothing. The approach is inspired by techniques presented in previous studies [52,53], which keep important, discriminative samples in the final training dataset and discard easily classifiable samples to rebalance the skewed ratio. Since the aim is to reduce the majority (negative) class, a sample is considered important (or ‘hard’) if it was incorrectly classified as a false positive. In order to ‘punish’ the false labeling of an instance, we utilized the probability, p, that the model misclassified a sample, and we introduced a novel instance hardness label smoothing approach, which changes the labels of samples to prevent models from producing overconfident predictions. Our approach first calculates the estimates of instance hardness (IH) on a model, or a committee thereof, to provide a better estimation of IH [54]. Subsequently, IH is smoothed out by being mapped from the range of [0,1] to the range of [0,0.5]. This ensures that the new, soft labels are not flipped and that for those samples that were the hardest to classify (an IH close to 1), the model is forced to be unsure rather than learn a new label for the given samples. miRBind was trained on the 1:1, 1:10, and 1:20 training sets, with the 1:20 model exhibiting the best performance.

#### 2.3.4. RNAhybrid

RNAhybrid [55] is, at its core, a variation of classic RNA secondary structure prediction. It determines the most favorable hybridization site between two sequences (a short and a long RNA) in a kind of domain mode. That means that the short sequence is hybridized to the best-fitting part of the long sequence. The method offers a web-service interface, as well as a standalone version. We evaluated the performance of the RNAhybrid by utilizing its standalone version with the default parameters.

#### 2.3.5. RNACofold

RNACofold [56] (henceforth mentioned as ‘cofold’) is a tool offered by the ViennaRNA package [57]. It computes the hybridization energy and base-pairing pattern of an input pair of interacting RNA molecule sequences. To simplify the performance comparison, minimum free energy scores were normalized to a range from 0 to 1, where 1 represents the strongest binding.

#### 2.3.6. RNA22

RNA22 [58] is a pattern-based approach for the discovery of microRNA binding sites and their corresponding microRNA/mRNA complexes that relies only on the sequences of miRNAs and their targets. The algorithm is based on a Markov chain that finds recurring patterns in miRNA sequences. Potential targets are then searched with the identified patterns, and areas with accumulated hits are paired with miRNAs, based on the nucleotide pairing and free energy. The standalone version of the RNA22 program was used and run with default parameters, apart from the ‘maximum folding energy for heteroduplex’, which was set to the maximum value of −5 kcal/mol. The score of the RNA22 was calculated as (1—*p* value), where *p* value is the output of RNA22 that characterizes each miRNA:target pair with which the method is predicted to interact. Each score was subsequently normalized by taking into account the minimum and maximum values of the scores.

#### 2.3.7. Seed

The ‘seed’ approach identifies a perfectly complementary match of the 2–7 miRNA hexamer on the target sequence. Since this is a binary decision, no area under the curve may be calculated.

#### 2.3.8. Web Interface

We built a publicly accessible, user-friendly web interface (https://ml-bioinfo-ceitec.github.io/miRBind/ (accessed on 28 November 2022) that allows for the prediction of the score (probability) of the binding between a user-submitted pair (or pairs) of miRNA and the target site. The web interface is implemented in HTML/CSS and JavaScript. The default model is trained on the CLASH dataset with a 1:1 positive:negative ratio. All relevant files and the default model are available on the miRBind GitHub repository.

#### 2.3.9. Evaluation Measures

For the assessment of classification tasks, a set of useful metrics are used. As has been demonstrated, the area under the precision-recall curve (auPRC) is the most informative visual analysis tool for highly imbalanced binary classification [59]. Sensitivity or recall is the proportion of true positive observations. Precision (Pre) is the ratio of true positive observations to the total number of predicted positive observations.

(1) $$\mathrm{Recall}=\frac{\mathrm{TP}}{\mathrm{TP}+\mathrm{FN}},$$

(2) $$\mathrm{Precision}=\frac{\mathrm{TP}}{\mathrm{TP}+\mathrm{FP}},$$

TP and FP are the numbers of true positive and false positive assessments, respectively. Additionally, TN is the number of true negative assessments.

## 3. Results

To evaluate our models, we followed a two-step process: first, we evaluated the performance of each of the DNN (miRBind, CNN, and DNABERT) for several positive:negative ratios of the validation sets, and selected the model that performs best among them. A complete overview of the evaluation of the models on the three ratios of the test sets is presented in Table 1. The models with the highest area under the precision-recall curve (auPRC) were the miRbind, CNN, and DNABERT models, trained on 1:20 and 1:10 and fine-tuned on 1:1 training sets, respectively.

**Figure 2:**
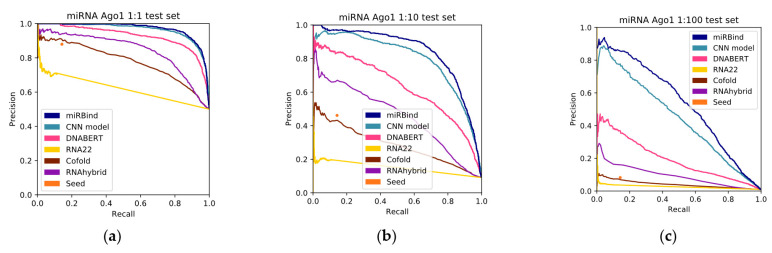
Precision-recall curves for all the methods, tested against (**a**) 1:1, (**b**) 1:10, and (**c**) 1:100 left-out test sets.

**Figure 3:**
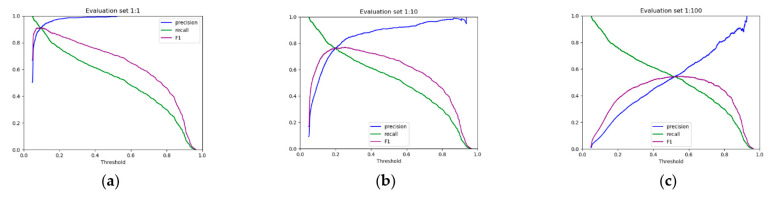
The precision, recall, and F1 score (the harmonic mean of precision and recall) against prediction score threshold for (**a**) 1:1, (**b**) 1:10, and (**c**) 1:100 datasets. The ‘normal’ (0.1) and ‘strict’ (0.5) score thresholds are suggested to users of miRBind.

Subsequently, we evaluated the performance of these best-performing models with the rest of the methods. To this end, we plotted the precision-recall curves (Figure 1) and we calculated the areas under the curves (Figure 2 and Table 2). As we can see in Table 2, miRBind outperforms ‘cofold’ with an AUPRC of 0.9689, vs. 0.7784 for ‘cofold’ with the 1:1 dataset. The difference is more pronounced in the more realistic 1:100 dataset, where miRBind100 shows an AUPRC of 0.5372 vs. 0.0413 for ‘cofold’. RNAhybrid exhibits better performance than the ‘cofold’ method, but this is still significantly lower than miRBind across all test sets, with its performance rapidly deteriorating with the increasing ratio of negatives. The ‘seed’ measure performs similarly to the co-fold method, showing a high precision score on the 1:1 balanced dataset, which has previously offered a promising method for miRNA target prediction programs, as well as for assigning miRNAs to CLIP-Seq peaks. Based on the trade-off between precision and recall at different prediction score thresholds (Figure 3), we have selected two score threshold cutoffs at 0.1 (‘normal’) and 0.5 (‘strict’) for general use.

The performance of the methods was also evaluated based on the area under the receiver operator characteristics curve (AUROC), as shown in Table 3.

**Figure 4:**
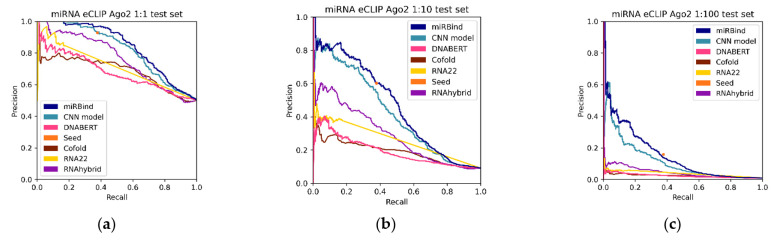
Precision-recall curves for all the prediction methods, tested against the (**a**) 1:1, (**b**) 1:10, and (**c**) 1:100 Ago eCLIP test sets.

To further investigate the predictive power of our approach, we evaluated its performance on the miRNA eCLIP dataset (Table 4). The precision-recall curves are presented in Figure 4. We validate that the miRBind model outperforms other methods in all imbalance categories. Our CNN model follows miRBind as the second-best method in all categories. It is interesting that the simple seed measure performs with much higher precision in this dataset than in the original CLASH data. Notably, in the 1:100 imbalanced dataset, the seed for the miRNA eCLIP dataset has double the precision and double the sensitivity than that for the CLASH dataset. This variation could point to differences between the seed-mediated binding affinities of Ago1 (CLASH) and Ago2 (miRNA eCLIP) proteins, or other experimental variations between the two experiments. In contrast, all other methods, including ours, seem to have a drop in performance between the two experiments.

## 4. Discussion

We have shown that the assignment of targets to miRNAs based on the ‘seed’ or ‘co-fold’ methods is unreliable in the highly imbalanced 1:100 scenario. Although these methods show over 90% precision in the balanced 1:1 scenario for a sensitivity/recall of 15–20%, in the 1:100 scenario, they produce nine false positives for each true positive they identify, yielding a precision of approximately 10%. In contrast, miRBind shows an almost perfect precision of up to 50% sensitivity/recall with the 1:1 balanced dataset and is more robust with the imbalanced 1:100 dataset. For a sensitivity of 50%, miRBind retrieves an approximately equal number of true positives and false positives. 

We have additionally validated our method on a completely new dataset of miR eCLIP, which was produced via a similar technique to the CLASH dataset used for training. The fact that our method outperforms the state of the art in such a different dataset further reinforces the theory that it has learned some rules of interaction between miRNAs and their targets and that this is not some experiment-specific bias. Our method was trained on CLASH data from the Ago1 protein and was tested on data from the miR eCLIP on the Ago2 protein. Even though the two proteins are considered to have similar modes of utilizing miRNAs to bind the targets, we see that the Ago2 dataset responds better to a simple seed prediction than the Ago1 dataset. We can infer that the Ago2 dataset is enriched in the canonical seed sequences, but we may not speculate if that represents a real difference between the Ago1 and Ago2 proteins, or just a difference between the CLASH and miR eCLIP methodologies. In principle, neither of these methods should enrich seed-based binding over other modes of binding. However, we cannot be confident as to whether some secondary effect of the experimental variation is in play.

To conclude, we presented two deep learning approaches for the prediction of miRNA:target binding, comprising a CNN that consists of six convolutional layers and a ResNet-based neural network. To avoid any potential target sequence or experimental biases, we elected to completely hide the sequences from the networks during training. To that end, we used a two-dimensional 50 × 20 representation of the miRNA and putative target sequence, in which any Watson–Crick binding nucleotide pair is represented by 1, and any non-binding pair by 0. To evaluate our approaches, we utilized three commonly used methods, namely, ‘seed’, RNAcofold, and RNA22. We also explored the capabilities of a pre-trained and fine-tuned DNABERT for the given task. We showed that our CNN and ResNet-based approaches outperform the state-of-the-art methods for miRNA:target site prediction.

We expect miRBind to be used by bioinformaticians interested in miRNA target prediction, as part of a larger pipeline, and by researchers interested in miRNA binding, for example, as a way to allocate miRNAs to CLIP-Seq peaks. For the first group of users that may want to run large numbers of pairs, we provide a standalone Python script with the miRBind method, which can be used locally on a CPU or GPU or may even be run on the freely available Google Colaboratory CPU and GPU. For the second group, who may not have the programming expertise that is needed, we provide a free web server at https://ml-bioinfo-ceitec.github.io/miRBind/ (accessed on 28 November 2022), which users can utilize to predict the potential binding between any miRNA-like sequence and any target sequence.

We believe that miRBind can be used to predict the pairing of miRNAs to their targets, improving on more basic methods. The fact that it has been trained without theoretical preconceptions beyond the Watson–Crick pairing makes it unbiased toward seed and non-seed bindings, an important feature that is needed to explain the increasing number of non-canonical binding sites that are being experimentally identified. One caveat of miRBind is that it was trained on Ago1 CLASH data, while Ago2 is the dominant Ago family protein involved with miRNAs. To date, no other Ago2 CLASH dataset has been published; as such, our validation dataset will be extremely important for future miRNA target-prediction methods. Additionally, the field is now open for further exploration of the differences between the Ago1 and Ago2 binding rules.

## Figures and Tables

**Table 1:** The area under the precision-recall curve (AUPRC) for the miRBind, CNN, and DNABERT models, tested against 1:1, 1:10, and 1:100 left-out test sets. The number used in the naming of each model indicates the positive:negative ratio on which the model was trained. We selected the best-performing miRBind and CNN models (miRBind20 and CNN10, henceforth called miRBind and CNN, respectively), as the proposed methods of our work.

AUPRC	Test Set 1:1	Test Set 1:10	Test Set 1:100
miRBind1	0.9495	0.7447	0.3079
miRBind10	0.9614	0.8092	0.4531
miRBind20	0.9689	0.8410	0.5372
CNN1	0.9602	0.7862	0.4095
CNN10	0.9634	0.7969	0.4464
CNN20	0.9590	0.7880	0.4365
CNN100	0.9599	0.8005	0.4466
DNABERT1	0.9267	0.6300	0.1923
DNABERT10	0.9250	0.6440	0.2286

**Table 2:** The area under the precision-recall curve for the miRBind, CNN, and DNABERT models, and the RNAhybrid, ‘cofold’, RNA22, and ‘seed’ approaches, tested against 1:1, 1:10, and 1:100 left-out test sets. The ‘seed’ method is evaluated based on its sensitivity and precision.

AUPRC	Test Set 1:1	Test Set 1:10	Test Set 1:100
miRbind	0.9689	0.8410	0.5372
CNN	0.9634	0.7969	0.4464
DNABERT	0.9267	0.6300	0.1923
RNAhybrid	0.8439	0.4539	0.0924
Cofold	0.7784	0.2842	0.0413
RNA22	0.6203	0.1507	0.0265
Seed	Sens: 0.1425 Prec: 0.8796	Sens: 0.1425 Prec: 0.4612	Sens: 0.1425 Prec: 0.0824

**Table 3:** Area under the receiver operator characteristics curve (AUROC) for the miRBind, CNN, and DNABERT models, as well as the RNAhybrid, ‘cofold’, RNA22, and ‘seed’ approaches, tested against 1:1, 1:10, and 1:100 left-out test sets. The ‘seed’ method is evaluated based on the false positive rate (fpr) and true positive rate (tpr).

AUROC	Test Set 1:1	Test Set 1:10	Test Set 1:100
miRBind	0.9643	0.9654	0.9652
CNN	0.9612	0.9626	0.9628
DNABERT	0.9293	0.9310	0.9310
RNAhybrid	0.8351	0.8406	0.8381
Cofold	0.7839	0.7839	0.7812
RNA22	0.5343	0.5342	0.5375
Seed	fpr: 0.0195 tpr: 0.1425	fpr: 0.0167 tpr: 0.1425	fpr: 0.0159 tpr: 0.1425

**Table 4:** The area under the precision-recall curve (AUPRC) for miRBind, CNN, and DNABERT models, as well as RNAhybrid, ‘cofold’, RNA22, and ‘seed’ approaches, tested against the 1:1, 1:10, and 1:100 Ago eCLIP test sets.

AUPRC	Test Set 1:1	Test Set 1:10	Test Set 1:100
miRbind	0.8413	0.4668	0.1545
CNN	0.8223	0.4268	0.1147
DNABERT	0.6787	0.1904	0.0238
RNAhybrid	0.7615	0.2932	0.0469
Cofold	0.6862	0.1946	0.0246
RNA22	0.7116	0.2628	0.0392
Seed	Sens: 0.3774 Prec: 0.9278	Sens: 0.3774 Prec: 0.6020	Sens: 0.3774 Prec: 0.1586

## Data Availability

All datasets and the full code for miRBind can be found at https://github.com/ML-Bioinfo-CEITEC/miRBind. The fully documented and publicly available pipeline for chimeric interaction detection is available at https://github.com/ML-Bioinfo-CEITEC/HybriDetector/. The miR eCLIP data are deposited with accession GSE218466.
